# Measuring Accuracy (Classification Probabilities, Positive, and Negative Predictive Values) of Executive Function Electroencephalogram Metrics in Attention-Deficit/Hyperactivity Disorder Diagnosis: Protocol for and Perspectives From the SINCRONIA Study

**DOI:** 10.2196/79150

**Published:** 2026-03-27

**Authors:** Hilario Blasco-Fontecilla, Javier Sánchez-Cerezo, Irene Gómez, Georgelina Abreu-Fernández, Sandra Ortiz, Jesús F Villoria, Miguel Blanco, Ana García, Julia Ballesteros, Roldán Martínez, Gerardo Gálvez, Fernando Maestú, Álvaro López-Medrano

**Affiliations:** 1 Department of Psychiatry, Emooti – Institute of Mental Health and Wellbeing, Calle José Abascal 51, Madrid, 28010, Spain, 34 910 149 575; 2 Instituto de Transferencia e Investigación (ITEI), Universidad Internacional De La Rioja (UNIR), Logroño, Spain; 3 Centro de Investigación Biomédica en Red de Salud Mental (CIBERSAM), Madrid, Spain; 4 Department of Psychiatry, Puerta de Hierro University Hospital, Majadahonda, Spain; 5 Bitsphi Diagnosis, Madrid, Spain; 6 Medicxact, Alpedrete, Spain; 7 Department of Experimental Psychology, Complutense University of Madrid, Madrid, Spain

**Keywords:** attention deficit hyperactivity disorder, ADHD, electroencephalogram, EEG, functional connectivity, machine learning, neural network, neurocognitive assessment, diagnosis, ADHD comorbidities.

## Abstract

**Background:**

Attention deficit/hyperactivity disorder (ADHD) is the most prevalent neurodevelopmental disorder worldwide, affecting approximately 5%‐7% of school-aged children and 2%‐5% of adults worldwide. However, there is still no reliable diagnostic tool for it. The lack of specific biomarkers further complicates the accurate diagnosis of ADHD.

**Objective:**

The SINCRONIA study seeks to develop and optimize an electroencephalogram (EEG)-based ADHD diagnostic classification algorithm by identifying biomarkers that provide optimal diagnostic performance.

**Methods:**

This protocol introduces a single-center, case-control study involving at least 165 participants, aged between 7 and 12 years, that is being conducted at the Puerta de Hierro University Hospital in Madrid, Spain. Participants will be allocated to 3 groups, including ADHD predominantly inattentive, ADHD predominantly combined or hyperactive/impulsive, and a control group, according to the best estimated diagnosis based on clinical interviews and a neuropsychological assessment that includes the Conners Continuous Performance Test. In addition, an EEG recording will be conducted separately, and functional connectivity metrics will be used to characterize brain networks associated with inhibitory control. The index test is expected to match or improve the clinical diagnosis of ADHD in children aged between 7 and 12 years and provide a set of eventual biomarkers that maximize diagnostic performance and provide pathophysiological clues.

**Results:**

The SINCRONIA study began screening and recruitment in March 2023. Recruitment ended on December 11, 2024. A total of 165 eligible participants were enrolled.

**Conclusions:**

The SINCRONIA project is a high-quality, large-scale, unicenter study devoted to improving the objective diagnosis of ADHD by using EEG biomarkers. The EEG-based ADHD diagnosis is expected to have greater sensitivity and specificity than the Conners Continuous Performance Test.

## Introduction

Attention-deficit/hyperactivity disorder (ADHD) is a neurodevelopmental disorder characterized by inappropriate levels of inattention, hyperactivity, or impulsivity, typically emerging in childhood with a relatively high prevalence [1]. Reliable estimates place the prevalence above 5% in children and adolescents and 2.5% in adults [2-5]. However, these rates vary by country, with the prevalence reaching 11.4% in American children aged 3-17 years in 2022, according to the Centers for Disease Control and Prevention [6].

ADHD is linked to neurocognitive deficits and impairments in psychosocial and vocational functioning, including increased mortality and suicide rates [7-12]. Neurocognitive deficits span a broad range of functions, including working memory, inhibitory control, and vigilance [13], as well as imbalances between immediate and delayed rewards [14], processing speed [15], and motor activation and control [16]. Furthermore, ADHD often co-occurs with other psychiatric disorders such as generalized anxiety, major depression, bipolar disorder, substance use, and conduct disorders; or with other neurodevelopmental disorders [17,18].

Accurate diagnosis is crucial for effective treatment planning and improving clinical research. Efforts to refine psychiatric nosology by exploring connections between neurobiology and psychopathology are underway [19-21] with a key advancement being the identification of biomarkers for psychiatric disorders [22,23]. Advances in understanding the pathophysiology of ADHD have highlighted alterations in noradrenergic signaling within posterior attentional circuits [24], disruptions in dopaminergic pathways across various systems [25], dysfunction of the default mode network [26], and delayed development of the frontal cortex. Despite these insights, little progress has been made in classification and diagnosis, as reflected by the fact that neither the *DSM-5* (*Diagnostic and Statistical Manual of Mental Disorders* [Fifth Edition]) nor the *ICD-11* (*International Classification of Diseases, 11th Revision)* grants a significant role to neurobiology.

Unfortunately, ADHD diagnosis remains inaccurate, partly due to its multifactorial etiology, psychiatric comorbidities, and neurocognitive and brain alterations. Although diagnosis is based on a comprehensive evaluation of current and past symptoms and functional performance, overlapping symptoms with other disorders—such as mood or learning disorders—and frequent co-occurrence with conditions like autism spectrum disorder (ASD), learning disabilities, personality disorders, or substance use further complicate differential diagnosis [27-31]. While some authors contend that ADHD has been overdiagnosed [32-35], others dispute this view [29,36]. Additionally, assessing the widespread impact of ADHD on various life areas with limited consultation time presents a challenge, requiring evaluation of multiple contexts [30].

ADHD is a dynamic condition where slowly developing neurobiological deficits are rapidly influenced by maturation and compensatory mechanisms, resulting in significant heterogeneity both across individuals and within the same individual over time, thereby complicating both diagnosis and treatment [37]. This heterogeneity has driven increased neuroimaging research and the search for biomarkers [38]. Functional magnetic resonance imaging (fMRI) studies have revealed delayed cortical development—especially in the frontal, temporal, and parietal regions—and disruptions in connectivity and default mode network functionality [39,40]. Moreover, deficits in working memory and inhibitory control have been linked to underactivation in frontoparietal and ventral attention circuits, which play key roles in executive function and attention reorientation [41]. Additionally, reduced activation in the ventral striatum has been associated with altered reward processing [42]. Research also indicates a loss of the typical counter-regulation between cognitive control and default mode networks during rest [36], with some studies suggesting compensatory hyperactivation in motor and visual areas [43]. Importantly, much of the observed heterogeneity may stem from the fact that ADHD subtypes or syndrome-level distinctions are often collapsed into single-group analyses, obscuring meaningful neurophysiological differences. Studies that stratify participants by ADHD subtype have begun to reveal distinct neural signatures and developmental trajectories, which may prove crucial for biomarker discovery and personalized intervention design [44].

These findings underscore the complexity of diagnosing and understanding attention disorders, highlighting the need for more precise and scalable diagnostic tools. In this context, electroencephalogram (EEG)-based techniques have gained increasing interest due to their ability to track dynamic interactions across large-scale brain networks with millisecond temporal resolution [45,46]. Unlike fMRI, EEG can directly measure neural activity and is better suited to capture transient cognitive processes, such as inhibition and attentional shifts, key domains affected in ADHD. Source-level EEG analyses further enhance spatial resolution, making it a practical and cost-effective alternative to higher-end techniques like magnetoencephalography in clinical research [47]. When applied to stratified samples reflecting distinct ADHD subtypes, EEG may help reveal subtype-specific connectivity patterns that are obscured in traditional binary comparisons. This supports its growing potential not only as a complementary biomarker platform but also as a tool for advancing precision diagnostics in neurodevelopmental disorders.

The SINCRONIA study builds on the 2 attention-control networks model developed by Corbetta et al [48]. The dorsal attention network (DAN) exerts top-down control by maintaining focus on task-relevant information while filtering out distractions, thus ensuring sustained attention. In contrast, the ventral attention network (VAN) evaluates each incoming stimulus based on its novelty and importance. When a stimulus is deemed high priority, the VAN activates—interrupting the DAN’s filtering process—and sends signals to trigger executive control, reorienting attention and prompting the appropriate response [49-51]. Conversely, if the stimulus is considered of low priority, the VAN deactivates, and the DAN’s inhibitory influence is reinforced, preventing unnecessary shifts in attention [48,52,53].

While structural brain alterations in ADHD are acknowledged [54], functional changes—reflecting the interactions within neural circuits—hold greater diagnostic relevance [30]. Although EEG’s diagnostic utility at the time of this writing is limited, its high temporal resolution, accessibility, and low cost support its potential in developing tools and identifying biomarkers [55,56]. Capturing transient neural dynamics has shown promise [57], and using multivariate methods with machine learning (ML) significantly enhances accuracy, especially when incorporating signal synchronization metrics [55]. However, existing EEG studies for ADHD detection remain suboptimal as diagnostic tools [58,59]. For all these reasons, the SINCRONIA study aims to develop an ML-based classification algorithm using EEG brain connectivity metrics and to determine the biomarkers that maximize the diagnostic performance on the Bit task (hereafter referred to as the index test).

The primary objectives of our study are:

1. To demonstrate that the diagnostic performance of the index test (EEG-based)—determined against the final diagnostic judgment of the study’s principal investigator (PI) following a comprehensive clinical assessment—exceeds a predefined minimum threshold of clinical acceptability.
2. To identify the biomarkers that optimize the diagnostic accuracy of the index test.

Secondary objectives are the following:

1. To develop and optimize a diagnostic classification algorithm based on selected biomarkers—identified in accordance with the primary objectives—to differentiate between controls, ADHD predominantly inattentive (without hyperactivity), and ADHD combined or hyperactive/impulsive (with hyperactivity) through classification models.
2. Investigation of factors influencing diagnostic performance, including patient demographics and clinical characteristics of ADHD, as well as health care provider factors (eg, specialty and experience).
3. To evaluate and compare the diagnostic performance of the index test—determined against the final diagnostic judgment of the study’s PI following a comprehensive clinical assessment—with that of the Continuous Performance Test Third Edition (CPT-3).
4. Assessment of the clinical utility and value of the index test.

Tertiary objectives are the following:

1. To investigate the construct validity of sluggish cognitive tempo (SCT) in comparison to ADHD diagnosis.
2. To optimize the preprocessing automatic system for artifact removal in EEG signals.
3. To explore Bayesian mathematical models and their application to brain functional connectivity patterns.

## Methods

### Study Design

This is a noninterventional, case-control, cross-sectional, and assessor-blinded study, which is part of the development of a medical diagnostic test. This manuscript describes the latest version of the study protocol (HUPH-BP-BMCCEEG-CC22 version 4.0, approved on June 7, 2024, as Amendment 4), which was reviewed and approved by the appropriate ethics committee (see below). In order to allow for more complete and detailed reporting, we adhere to 2 acceptable reporting guidelines that match the study design: the Standards for Reporting Diagnostic Accuracy Studies (STARD; Checklist 2) and the SPIRIT (Standard Protocol Items: Recommendations for Interventional Trials) 2025 checklist (Checklist 1) of items to address in a randomized trial protocol, though, *sensu stricto*, our study is not a randomized trial. Following the terminology of the STARD, the EEG and the algorithms used to analyze the EEG data will be regarded as the index test, and the executive control task during which brain connectivity metrics will be pursued will be regarded as the index task. The best estimated diagnosis based on clinical interviews and neuropsychological assessment will be regarded as the reference test. To minimize bias, the PI establishing the reference standard will not perform EEG analyses or access EEG results. Blinding to index test results will be maintained, as no diagnostic or therapeutic decisions will be based on these findings. Consequently, no clinical decision will be influenced by information from the index test. Likewise, the team in charge of EEG processing will not have access to the clinical diagnosis data during data collection for the study. Participants will be categorized into 3 groups, including ADHD predominantly inattentive, ADHD combined or hyperactive/impulsive, and a control group, based on clinical evaluation including the reference CPT-3.

### Participants

A total of at least 165 participants will be recruited (Multimedia Appendix 1). The target population will consist of children who exhibit symptoms and signs suggesting ADHD who contact the health system and who may or may not have the disorder. It will be attempted to represent this population through a source population consisting of (1) children aged 7-12 years who either received a prior diagnosis of ADHD (cases; predominantly hyperactive/impulsive or combined presentation or predominantly inattentive) or (2) children attending routine pediatric check-ups at the Puerta de Hierro University Hospital in Majadahonda (HUPHM) who do not meet criteria for any significant psychiatric disorder (controls). Recruitment ended in December 2024. Accordingly, our study was a convenience series of participants.

A data monitoring committee was not considered necessary due to the low-risk nature of the study; oversight is ensured through standard monitoring procedures.

### Instruments

#### Tanner Scale

The Tanner sexual maturity scale will be administered to assess the degree of development and physiological status in participants [60].

#### Strengths and Difficulties Questionnaire

The Strengths and Difficulties Questionnaire (SDQ) is a brief screening questionnaire that helps detect pediatric psychopathology in individuals between 4 and 16 years old, with measures of social, emotional, and behavioral functioning. It consists of 5 scales (emotional symptoms, conduct problems, hyperactivity/inattention, peer relationship problems, and prosocial behavior) and provides a total score of difficulties. Due to its use in scientific literature and the demonstration of its good psychometric properties, this scale has been used to rule out relevant psychiatric pathology that could interfere with the study [61,62].

#### Child and Adolescent Behavior Inventory

The Child and Adolescent Behavior Inventory (CABI) consists of 9 subscales that allow measuring symptoms of inattention, hyperactivity, SCT, anxiety, depression, oppositional defiant disorder, prosocial attitude, and academic and social difficulties. The parent version will be used for this study. Each scale is made up of several items, and it must be specified whether the absence or presence of such symptoms has an impact on an academic or social level (no difficulty, mild difficulty, moderate difficulty, or severe difficulty). We will use the most recent normative data that have been published for Spanish youth aged 5‐16 years [63].

#### ADHD Rating Scale IV

The validated Spanish version of the Attention Deficit/Hyperactivity Disorder Rating Scale IV (ADHD-RS-IV) will be used to check the severity of ADHD, considering the total score of the scale—composed of 18 items that are scored according to a 4-point Likert scale (0=never or rarely, 1=sometimes, 2=often, or 3=very often)—and the scores of the 2 subscales of the test, inattention and hyperactivity/impulsivity [64].

#### Wechsler Intelligence Scale for Children, Fifth Edition

The Wechsler Intelligence Scale for Children, Fifth Edition (WISC-V) allows for the assessment of the total IQ and consists of 5 main indices (verbal, visuospatial, fluid reasoning, working memory, and processing speed) that are measured from 10 tests. Secondary indices (quantitative, auditory working memory, nonverbal, general ability, and cognitive competence) will also be included [65].

#### Continuous Performance Test

The CPT-3 assesses attention, impulsiveness, and processing speed through a go/no-go interference task where the participant must press the space bar on the keyboard to all letters that appear on a computer screen, except for the letter X (nontarget). The letter presentation interval is 1, 2, and 4 seconds, and the test measures omission errors (unanswered targets), commission errors (answered nontargets), hit reaction time, and response speed variability. The test lasts for 14 minutes [66]. The test positivity cutoff is automatically generated by the program depending on age and sex of the participant.

#### Neuropsychological Evaluation of Executive Functions

The Neuropsychological Evaluation of Executive Functions (ENFEN) is an assessment battery applicable to children between 6 and 12 years old that consists of 4 tests (fluency, paths, rings, and interference) that measure executive functions. The full battery will be used in this study [67].

#### Test for Detecting Dyslexia in Children

For this study, a screening battery consisting of 12 tests known as PROLEXIA (Emooti Neurotech SL) will be used. These tests are applicable from the age of 7 years and are designed to detect symptoms of dyslexia, particularly those related to the phonological component, aiding in differential diagnosis [68].

#### Child Concentration Inventory-Version 2

The Child Concentration Inventory-Version 2 (CCI-2) is a test developed to detect SCT. It is designed to be self-administered by children. It is composed of 16 items, rated on a 4-point Likert scale (0=never, 1=sometimes, 2=often, or 3=always) [69].

### Index Test

The index test (also known as the Bit task) is a go/no-go interference test consisting of 3 parts, preceded by a 4-minute resting-state EEG recording. Each part of the test contains 400 trials, with an interstimulus interval between 450 and 550 milliseconds long. The ratio of go and no-go trials changes across parts, including 25:75 (first part), 50:50 (second part), and 75:25 (third part). If participants make mistakes, a warning will be displayed on the screen reminding them of the instructions. This task will be executed using PsychoPy software (version 2022.1.3; Open Science Tools Ltd).

### EEG Recording System

We will use the Waveguard net (ANT Neuro) HD-C68 Tyco with Ag rings, 2 HD-C68pin connectors, and 64 semidry electrodes arranged in an equidistant layout, compatible with eego processing software (version 1.9.2 or higher; ANT Neuro). The EEG eego adapter will be used for 6 type A channels, along with the eego amplifier EE-225 (ANT Neuro BV). The reference signal is placed at the 5Z position, while the ground signal is positioned at the 0Z coordinate.

### Participant Recruitment

The study will be conducted in the Department of Psychiatry at the HUPHM. Participants will be recruited from 2 distinct sources. First, cases will be identified by reviewing medical records from the hospital to locate participants with an existing diagnosis of predominantly inattentive, predominantly hyperactive/impulsive, or combined ADHD prior to the commencement of study procedures. Additionally, cases may be prospectively selected from patients routinely seen at the study site who meet the specified selection criteria. These diagnoses will be confirmed through clinical evaluation during the study reference test. Control participants will be recruited from individuals attending Pediatric Services at HUPHM.

Selected candidates will be contacted and invited to participate, with an in-person appointment scheduled to complete the study procedures. ADHD cases will be instructed to temporarily discontinue their medication for the disorder prior to the first visit, with a 24-hour cessation for those using methylphenidate or atomoxetine and 48-hour for those using lisdexamfetamine.

### Study Visits

#### First Visit

During the first visit, the study’s purpose will be thoroughly explained to participants. Following this, informed consent will be obtained from parents or guardians, and assent will be sought from the child participants. No data will be collected if the participant withdraws consent from the study. The eligibility criteria (Table 1) will be reviewed to confirm participants’ suitability. Additionally, participants will be instructed to abstain from stimulant substances, such as caffeine, xanthines, or medications, 24-48 hours prior to the visit, depending on the type of treatment. If this time frame has not been adhered to, the study evaluation will be rescheduled for the following days.

**Table 1.** Eligibility criteria for participants in the SINCRONIA study.

Participant group	Inclusion		Exclusion	
	Cases (ADHD^a^)	Pediatric controls (non-ADHD)	Cases (ADHD)	Pediatric controls (non-ADHD)
Agreement to participate, with written informed consent obtained from a legal representative together with their own assent	Yes	Yes	No	No
Age (years)	7-12	7-12	<7->13	<7->13
Right-handedness (recruitment of up to 10% of left-handed children is permitted)	Yes	Yes	No	No
Time schedule	ADHD diagnosis within the past 3 years or at the time of the study	Pediatric attendance within the past 3 years or at the time of the study	ADHD diagnosis more than 3 years before the time of the study	Pediatric attendance more than 3 years before the time of the study
Type of ADHD	Predominantly hyperactive/impulsive, or combined presentation, or predominantly inattentive presentation	—^b^	ADHD presentations different from those considered in the inclusion criteria	—
Neuropsychiatric comorbidities	Mild/moderate comorbid conditions (such as anxiety, dyslexia, or SCT^c^) that do not compromise participation	Mild/moderate comorbid conditions (such as anxiety, dyslexia, or SCT) that do not compromise participation	Severe comorbid conditions (such as autism or epilepsy) that may compromise participation	Severe comorbid conditions (such as autism or epilepsy) that may compromise participation
Color blindness or any other condition that may cause instrumental difficulties for sensorineural perception	No	No	Yes	Yes
Severe cognitive or behavioral problems or insufficient capacity to maintain a minimum level of relaxation and rest	No	No	Yes	Yes
Regular intake of medications other than the treatment of ADHD that may alter attentional or executive capacity	No	—	Yes	—

a ADHD: attention-deficit/hyperactivity disorder.

b Not applicable.

c SCT; sluggish cognitive tempo.

Sociodemographic and clinical data will be collected from eligible participants, including a physical assessment to document their maturation status (Tanner stage) and a record of all prior treatments at the time of this writing, including those for ADHD. Questionnaires will be administered to screen for relevant psychiatric disorders (SDQ) and comorbid conditions (CCI-2 and CABI). The WISC-V will be conducted as part of the neuropsychological evaluation. If the assessment is not completed during the first visit, an additional visit will be scheduled to complete the evaluation.

#### Second Visit

During the second visit, the suspension of medication and abstinence from stimulant substances such as caffeine and xanthines will be confirmed with the participants. Once both have been confirmed, the child’s head circumference will be measured to select the appropriate EEG cap size (S, M, or L). The cap will then be immersed in saline solution for 10 minutes before being placed on the child’s head. The impedance measured in ohms will be assessed at this point.

The EEG recording will begin with a trigger check and a 4-minute eyes-closed resting phase to record brain activity at baseline, followed by the task. The instructions for the task will be introduced, with example trials before full implementation of each part of the task. Throughout the task, breaks will be provided for brief play activities and impedance checks. The EEG session will conclude with the creation of a 3D model of cap placement while the child is wearing the cap.

#### Third Visit

During the third visit, the suspension of medication and abstinence from stimulant substances such as caffeine and xanthines will be again verified. Once both are confirmed, the neuropsychological evaluation will proceed with the administration of the CPT-3, the ENFEN, and additional tests to screen for comorbid conditions, such as PROLEXIA.

**Figure 1.**
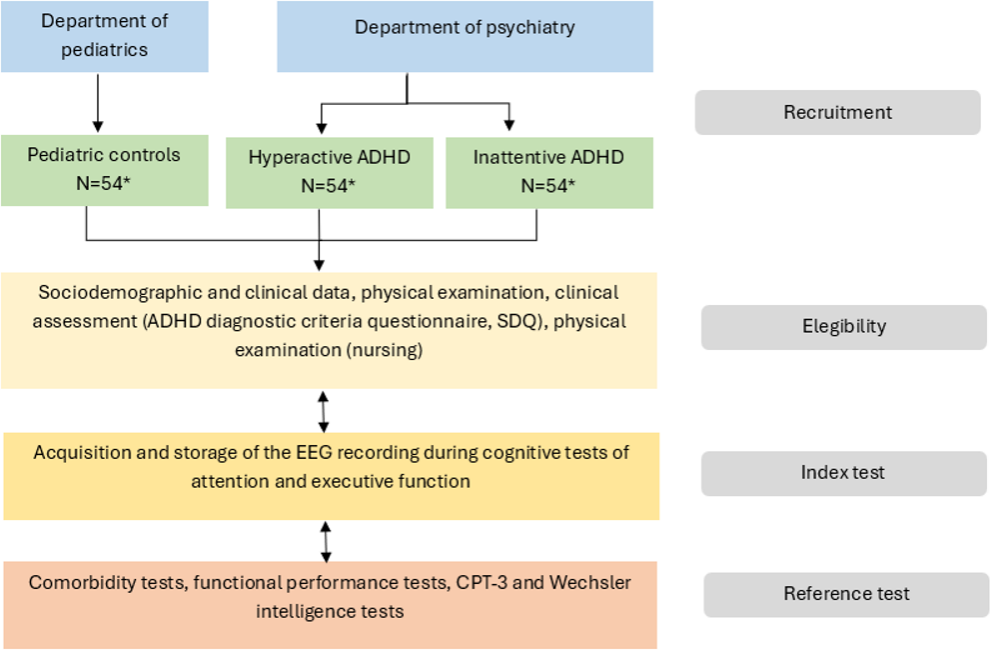
Procedure of the study. ADHD: attention deficit/hyperactivity disorder; CPT-3: Continuous Performance Test, Third Edition; EEG: Electroencephalogram; SDQ: Strengths and Difficulties Questionnaire.

Although the sample size calculation indicates a total of 120 participants (40 per group), an additional 10 participants per group will be recruited to explore factors influencing diagnostic performance and SCT construct validity (Figure 1). Furthermore, an extra 10% will be added to account for nonassessable data, resulting in a total of 162 participants. This total may be slightly increased to ensure 54 participants per group after accounting for screening failures and protocol deviations. The maximum time interval between the index test and the reference standard is 72 hours; however, whenever feasible, a 24-hour interval will be pursued.

### Planned Data Analysis

This clinical study focuses on the characterization and initial validation of an experimental medical diagnostic test under development. To avoid bias, the physicians involved in diagnosing and selecting the study groups will not participate in the analysis. Furthermore, missing data will not be imputed. In addition, indeterminate index test or reference standard results will not be allowed, as a positive or negative diagnosis will be required according to the previously defined cutoffs. Analyses will be performed on complete cases within each analysis set. The planned sample size (Multimedia Appendix 1) includes a margin for nonevaluable data (10% added for nonassessable data-complete-case analysis). No interim analyses or stopping rules are planned.

#### Hypotheses

The primary hypothesis, though involving 3 participant groups, focuses on demonstrating a minimum diagnostic performance to distinguish between the ADHD group as a whole and the pediatric control group. Regarding the true positive fraction (true positive fraction [TPF] or sensitivity), due to its high acceptability threshold (TPF_0_=0.90), a noninferiority test will be used for this dimension, with a noninferiority margin of δT_0_=−0.10. Since the primary objective is to determine whether the index test yields classification probabilities—TPF and false positive fraction (false positive fraction [FPF] or 1-specificity)—that are minimally acceptable (TPF_0_ and FPF_0_), the corresponding hypothesis, considering the noninferiority assumption for sensitivity, can be formally stated as:


(1) $$H_{0}:\left\{TPF-TPF_{0}\leq \delta \;\overset{T}{\underset{0}{\cup }}FPF\geq FPF_{0}\right\}$$

If H_0_ can be rejected, TPF−TPF_0_ > δ$\begin{array}{c} T\\ 0 \end{array}$ and FPF<FPF_0_; therefore, the index test would be noninferior to a hypothetical test with 90% sensitivity and superior to a hypothetical test with a specificity of 1−FPF_0_.

Two additional pairwise comparisons will be conducted: one between each ADHD group and pediatric controls, and another between the ADHD groups themselves, with hypotheses formulated as previously described. The Hochberg procedure will be used to control Type I error [70].

Biomarker identification will use dimensionality reduction methods, which are descriptive and do not require hypothesis testing or multiplicity adjustments.

#### Statistical Analysis

The primary objective will be to assess the diagnostic accuracy of the index test in terms of classification probabilities, specifically sensitivity and specificity. Sample size calculation ensures a minimum performance threshold within the target population.

This objective is interdependent with another primary aim, identifying the minimal set of biomarkers that optimize the diagnostic performance of the index test. As this objective does not involve hypothesis testing, adjustments for multiplicity are unnecessary.

The analyses of the secondary objectives will be exploratory, except for the first, which shares the hypothesis-free nature of biomarker identification. Tertiary objectives will also be exploratory, focusing on the validity of the SCT construct and EEG signal preprocessing systems. These analyses may be expanded in future studies to identify EEG-based clusters associated with ADHD symptomatology.

#### Analysis of Primary Parameters

The analysis of diagnostic accuracy requires preliminary analyses to identify biomarkers that optimize the diagnostic performance and to develop a classification algorithm based on biomarker values across the three study groups defined by the clinical team, serving as the reference test. The first secondary parameter, essential for evaluating the primary parameter, will also be analyzed in a confirmatory fashion without explicit hypothesis testing.

#### Diagnostic Performance of the Index Test

The primary parameters are the classification probabilities of the index test relative to the reference test. In addition to these classification probabilities, positive and negative predictive values (PPV and NPV, respectively) and diagnostic likelihood ratios will be calculated. Inferences will be made by calculating 95% asymmetric rectangular confidence regions for each pair of interest values (TPF and FPF), (PPV and NPV), and (DLR^+^ and DLR^−^). Confidence regions will be calculated from the cross-product of the 97.5% confidence intervals of each component in each pair. Sensitivity values will be assessed for noninferiority (and potentially superiority), while specificity will be evaluated for strict superiority. In addition to naive predictive value estimates, which will be biased by design, other estimates will be adjusted using external prevalence data and diagnostic likelihood ratios or Bayesian multiplication factors to mediate prior and posterior probabilities:

, (2) $$logit\;PPV=logit\;\rho +\log{DLR}^{+}$$

, (3) $$logit\;NPV=-logit\;\rho \;+\;-\log{DLR}^{-}$$

where ρ is the prevalence in the target population (7.0% [71]). Clinical information and reference standard results will not be available to the performers of the index test. Furthermore, the reference standard will be performed after the index test. Moreover, performers of the index test will neither be involved in the technological processing nor the statistical analyses of either the index or the reference standard tests. Finally, cross-tabulation of the index test results (or their distribution) by the results of the reference standard will be considered.

### Ethical Considerations

This study was approved by the Research Ethics Committee of the HUPHM on February 13, 2023 (PI 207/22). Informed consent was obtained from legal guardians and assent from minors, in accordance with the provisions of Organic Law 3/2018 of December 5 [72], on the Protection of Personal Data and Guarantee of Digital Rights. All procedures involving human participants were conducted in accordance with the ethical standards of the responsible institutional and national research committee and with the 1964 Declaration of Helsinki and its subsequent amendments. Monitoring of the clinical trial was carried out as required by applicable clinical research regulations. Privacy was always guaranteed. All participants had a secret code which was dissociated and saved separately from personal identification data. All participants entered into the study without compensation. All data generated or analyzed will be fully anonymized to ensure participant confidentiality (ISRCTN Registry ISRCTN12110752).

### Risks and Adverse Events

Any adverse events from the EEG recordings, index task, and neuropsychological tests will be recorded according to the clinical guidelines established in the legal framework. Adverse events will be recorded from the first visit to the last. The researcher (HB-F) will assess the association between the events and the assessment and report this to the research ethics committee.

### Data Management

Data will be collected using a pre-established standardized variable form and entered in a password-protected electronic database with role-based access control. Data quality will be ensured through predefined regular monitoring and periodic audits to identify missing or inconsistent entries. Access to the final dataset will be restricted to authorized members of the research team.

## Results

The SINCRONIA study began screening and recruitment in March 2023. Recruitment ended on December 11, 2024. A total of 165 eligible participants were enrolled. This research was funded by the sponsor, Bitsphi Diagnosis. The SINCRONIA study was registered in the ISRCTN Registry, a primary clinical study registry recognized by the World Health Organization (Trial registration: ISRCTN12110752; February 20, 2025).

## Discussion

### Comparison With Prior Work

EEG has been extensively employed to explore various neurophysiological metrics in ADHD, such as power spectral densities and event-related potentials. For instance, Clarke et al [73] demonstrated that children with ADHD exhibit increased theta band activity, decreased alpha band activity, and an elevated theta/beta ratio (TBR) compared to controls. Furthermore, these differences persisted into adulthood, with individuals showing lower beta levels in specific regions and a reduction in the ratio in ADHD compared to controls in recordings with eyes closed [74]. Similarly, Jarret et al [75] found increased relative theta power in the T5 and F7 regions in individuals with ADHD, aligning with findings from Chutko et al [76], who observed elevated TBR in children with both ADHD and SCT. Despite these promising findings, the utility of metrics like TBR as a robust diagnostic tool has been questioned due to inconsistent results [77,78], although in recent years researchers have increasingly favored the use of TBR in combination with Bayesian Gaussian models [79].

Although EEG power metrics offer valuable insights, recent studies have shifted focus toward functional connectivity analyses, which may serve as more stable and reliable biomarkers for ADHD. McNorgan et al [80] identified impairments in functional connectivity within the default mode network, reinforcing the hypothesis that ADHD involves disrupted brain networks underlying attention and executive control. Michelini et al [81] further highlighted abnormalities in the corpus callosum and connectivity between the default mode network and frontoparietal/salience networks, suggesting that ADHD is fundamentally a disorder of brain connectivity and providing critical insights by examining brain connectivity across controls, individuals with persistent ADHD, and those in remission. Their findings revealed that individuals with ADHD exhibited less pronounced increases in theta band connectivity following cognitive control tasks, indicating deficits in modulating connectivity during transitions from rest to task-oriented states. Additionally, during the Flanker task, those with ADHD demonstrated global hyperconnectivity in theta, alpha, and beta bands when the task’s prestimulus cues were incongruent and only showed hyperconnectivity in the beta band during the processing of target stimuli when the stimuli were congruent, which correlated with poorer cognitive control and dysfunctional behavior [81]. These findings are consistent with previous research showing enhanced connectivity in the alpha and beta bands during attention tasks [36,82].

Despite recent advancements, significant limitations persist in the literature. Many studies rely on small sample sizes and predominantly male samples or fail to report sex distribution, limiting the generalizability of their findings [81,83,84]. Furthermore, individual biomarkers often explain only a small fraction of the variance in treatment responses, highlighting the need for multifactorial and integrative approaches.

In addition, several methodological issues remain unaddressed in previous studies. Most existing tasks used for biomarker discovery are not grounded in theoretical models of attentional functioning and do not incorporate inhibition as a core element of the pathology, despite its central role in ADHD-related executive dysfunction [7]. These studies also tend to overlook functional connectivity at the source level and instead rely on scalp-level analyses, which limits the spatial precision and neural interpretability of their findings [45]. Moreover, EEG-based investigations are rarely validated against higher spatial resolution techniques such as magnetoencephalography, which could serve as a useful benchmark for signal source validation [47]. Finally, many studies use binary group comparisons (ADHD vs controls), neglecting the added value of including subtypes or differential diagnoses such as SCT and dyslexia in a 3-group design, which would allow for more clinically informative models [44,85].

Our study targets to address these gaps by using functional connectivity patterns, derived from EEG signals in the frequency domain, as biomarkers for ADHD. In this study, which will include at least 165 participants, we seek to improve the diagnostic accuracy achieved in the clinic at the time of this writing and to differentiate ADHD subtypes or other comorbid conditions that might be potentially confused with ADHD, using EEG-based ML algorithms applied to a go/no-go task. This study’s strength lies in its comprehensive design and robust sample size, enabling the identification of ADHD with high accuracy.

By integrating EEG connectivity metrics with ML algorithms, we aim to uncover reliable biomarkers for ADHD, advancing the field and contributing to a deeper understanding of this neurodevelopmental disorder.

### Strengths and Limitations

The study included primarily right-handed children (10% left-handed) aged 7‐12 years who could suspend medication and tolerate EEG recording sessions. The inclusion of left-handed children, up to 10% of eligible participants (Table 1), partly addressed this limitation within the study. Despite excluding children with ASD or other comorbidities, the sample of this study is fairly representative of most ADHD populations. However, the team is planning future studies to examine biomarker performance in more heterogeneous, clinically representative ADHD populations, including children with common comorbidities, such as ASD.

Another limitation was that the final clinical diagnosis made by the PI was used as the reference test, as well as to define the participant grouping for algorithm training, thus introducing some circularity that could inflate diagnostic accuracy. However, several measures were introduced in order to mitigate this issue: first, the PI established the diagnosis independently, following established clinical criteria and without knowledge of any algorithmic or EEG outputs, thereby reducing the risk of circularity; second, several patients were previously diagnosed by other professionals, and the PI only confirmed the ADHD diagnosis; and third, a contract research organization was hired and an external neuropsychologist independently evaluated all patients.

Despite the aforementioned limitations, the study also presents notable strengths. First, it benefits from a large sample size, which enhances the reliability of the findings and supports the generalization of the results and allows for comparisons between ADHD subtype groups. Additionally, it incorporates a comprehensive neuropsychological assessment that enables a detailed examination of participants’ cognitive profiles. This approach allows for a more accurate identification of the specific neurocognitive processes affected in children with ADHD, providing a robust dataset that can inform future research and support the exploration of other clinical and neurodevelopmental aspects of the disorder across different ages.

## Supplementary material

10.2196/79150 Multimedia Appendix 1 Sample size determination.

10.2196/79150 Checklist 1 SPIRIT checklist.

10.2196/79150 Checklist 2 STARD checklist.

## Abbreviations

ADHD: attention deficit/hyperactivity disorder
ADHD-RS-IV: Attention Deficit/Hyperactivity Disorder Rating Scale IV
ASD: autism spectrum disorder
CABI: Child and Adolescent Behavior Inventory
CCI-2: Child Concentration Inventory-Version 2
CPT-3: Continuous Performance Test, Third Edition
DAN: dorsal attention network
*DSM-5*: *Diagnostic and Statistical Manual of Mental Disorders, Fifth Edition*
EEG: electroencephalogram
ENFEN: Neuropsychological Evaluation of the Executive Functions
fMRI: functional magnetic resonance imaging
FPF: false positive fraction
HUPHM: Puerta de Hierro University Hospital in Majadahonda
*ICD-11*: *International Classification of Diseases, 11th Revision*
ML: machine learning
NPV: negative predictive value
PI: principal investigator
PPV: positive predictive value
SCT: sluggish cognitive tempo
SDQ: Strengths and Difficulties Questionnaire
SPIRIT: Standard Protocol Items: Recommendations for Interventional Trials
STARD: Standards for Reporting Diagnostic Accuracy Studies
TBR: theta/beta ratio
TPF: true positive fraction
VAN: ventral attention network
WISC-V: Wechsler Intelligence Scale for Children, Fifth Edition
