# Supplementary material for: Measuring Accuracy (Classification Probabilities, Positive, and Negative Predictive Values) of Executive Function Electroencephalogram Metrics in Attention-Deficit/Hyperactivity Disorder Diagnosis: Protocol for and Perspectives From the SINCRONIA Study
Source: JMIR Res Protoc. 2026 Mar 27;15:e79150. doi: 10.2196/79150 (PMC13033445; doi:10.2196/79150)
Supplement: Multimedia Appendix 1 [file resprot-v15-e79150-s001.docx]

Sample Size Determination

This is a Multimedia Appendix to a full manuscript published in the J Med Internet Res. For full copyright and citation information see <http://dx.doi.org/10.2196/jmir.xxxx>. Sample size was estimated using asymptotic formulas for comparing binomial proportions, reflecting the categorical outcomes of the index and reference tests. No adjustments for regression-based contrasts are planned, as factor analysis will be exploratory. An additional 10 participants per group will be included to support differential diagnosis analyses, particularly for dyslexia and SCT.

To test the hypothesis (equation 1) considering the values y indicated in the previous section, and FPF_0_=0.70, TPF=0.96, and FPF=0.90 for the minimum threshold of acceptability for specificity and the expected classification probabilities, respectively, the composite hypothesis H_0_ will be tested ensuring that the rectangular 1 − α* confidence region of both parameters in the (TPF, FPF) plane is above the values TPF_0_ and FPF_0_ with probability 1 − β*. If α=.05 and β=.20, then and to ensure the error margins. Since power can be expressed in terms of the limits of the 1 − α* confidence region, lower for TPF () and upper for FPF (), as:

, (2)

Using asymptotic expressions for variance, the following formulas can be derived for the number of participants with the disorder under study and pediatric controls:

(3)

(4)

Where Z^1−α*^ and Z^1−β*^ represent the corresponding quantiles of the standard normal distribution ( and , respectively). Substituting the values indicated above into (equation 3) and (equation 4), we obtain and . Consequently, and considering a 10% reserve for nonevaluable data and an additional 10 participants per group, a total of 165 participants will be recruited for the study, with 54 participants in each of the 3 groups. The previously mentioned Hochberg adjustment procedure does not require inflation of the sample size.

Strategies for achieving adequate participant enrollment to reach target sample size included publicizing the study in our Hospital.

| H_0_: {TPF − TPF_0_ ≤ δ ∪ FPF ≥ FPF_0_} | () *eq. 1* |
| --- | --- |
| Logit PPV=logit ρ + log DLR^+^, | (eq. 5) |
| logit NPV = −logit ρ + −log DLR^−^, | (*eq. 6*) |
